# High-Frequency Transcutaneous Electrical Nerve Stimulation in the Management of Pyramidal Tract-Related Spasticity: A Systematic Review

**DOI:** 10.7759/cureus.86298

**Published:** 2025-06-18

**Authors:** Athanasios K. Chasiotis, Vasileios Giannopapas, Marianna Papadopoulou, Thomas Panagopoulos, Dimitrios Stasinopoulos, Sotirios Giannopoulos, Daphne Bakalidou

**Affiliations:** 1 Department of Physiotherapy, University of West Attica, Athens, GRC; 2 Second Department of Neurology, Attikon University Hospital, National and Kapodistrian University of Athens, Athens, GRC

**Keywords:** pyramidal tract, pyramidal tract-related spasticity, spasticity, transcutaneous electrical nerve stimulation or high-frequency transcutaneous electrical nerve stimulation, upper motor neuron syndrome

## Abstract

It is known that the pyramidal tract is the main pathway that carries signals for voluntary movements. In upper motor neuron lesions, lesions to the pyramidal tract can lead to devastating consequences, one of which is spasticity. Among other rehabilitation techniques, the use of high-frequency transcutaneous electrical nerve stimulation (HF-TENS) could be beneficial in spasticity management. The goal of this systematic review was to summarize previously published information on the use of HF-TENS in the management of pyramidal tract-related spasticity (PTrS). A thorough research of the PubMed, MEDLINE, and Scopus databases was performed. The search identified 340 records. After screening, nine records met the inclusion-exclusion criteria and were assessed. The included studies investigated the effectiveness of HF-TENS on pyramidal tract-related lower limb spasticity. Spasticity was measured through the Modified Ashworth Scale (MAS), Composite Spasticity Scale (CSS), and spinal inhibition reflexes through the H-reflex. Seven records used a 30-minute HF-TENS in stroke human patients with lower limb spasticity. Results showed post-TENS reduction in spasticity and enhancement in balance ability without any significant alteration in Hoffmann's reflex (H-reflex) (p < .05). The last two records performed HF-TENS in multiple sclerosis and spinal cord injury patients with lower limb spasticity. The findings showed that 60-minute HF-TENS alleviated spasticity levels and pain levels as well as decreased resistance to full range of motion (ROM) and ankle clonus (p < .05). HF-TENS seems to be a promising therapeutic tool in managing PTrS. However, there is a need for homogenization of application parameters in order to be applied in rehabilitation centers.

## Introduction and background

The pyramidal tract is considered the primary pathway that is responsible for transmitting signals of voluntary movements [1]. In upper motor neuron syndrome (UMNS), damage to the pyramidal tract can have profound consequences, one of the most significant being spasticity, a motor disorder characterized by a velocity-dependent increase in tonic stretch reflexes and exaggerated tendon jerks. Due to the loss of inhibition in lower motor neuron pathways, spasticity is considered a "positive" manifestation of UMNS, which is attributed to a sensory-motor control deficit within the muscle regulation system. Furthermore, in UMNS lesions, hypertonic flexion patterns typically present in the upper limbs, while hypertonic extension patterns are observed in the equilateral lower limb. A common manifestation of pyramidal tract-related spasticity (PTrS) is electric, lateralized sensory and motor dysfunction [1].

Spasticity manifests across a broad spectrum of conditions and varies in severity [2]. The most common neurological conditions characterized by UMNS are 35% of stroke patients, 50% of traumatic brain injury (TBI) survivors, 40% of individuals with spinal cord injury (SCI), and between 37% and 78% of patients with multiple sclerosis (MS) [2]. Spasticity’s consequences are profound and include symptoms such as pain, urinary dysfunction, excessive fatigue, insomnia, as well as reduced mobility and loss of functionality. All these factors have been associated with a decline in overall health, which can lower the patients’ quality of life significantly [3].

In the UMNS patients’ rehabilitation strategy, one intervention that is widely used for pain management is transcutaneous electrical nerve stimulation (TENS). TENS is a noninvasive neuromodulation technique that targets large sensory fibers by activating specific natural pain relief mechanisms through the Pain Gait Mechanism and the Endogenous Opioid System. According to the pain gait mechanism, activation of the Aβ fibers reduces the transmission of the noxious stimulus from the "c" fibers through the spinal cord and hence onto the higher centers. The Aβ fibers are being stimulated at a relatively high rate (≥90 Hz) [4]. TENS is typically administered through adhesive electrodes, allowing for adjustments in parameters such as frequency, intensity, and duration. TENS application varies between high-frequency TENS (HF-TENS) and low-frequency TENS (LF-TENS) [4]. Low-frequency modes are defined as being 10 Hz or less, while high-frequency modes are typically described as ranging up to 50 or 100 Hz and above [2,3]. In addition, HF-TENS activates small-diameter, high-threshold cutaneous afferents (A-delta) in order to inhibit peripheral nerve nociceptive information transmission and to activate mechanisms of extrasegmental analgesia [3]. Given the recent advances in the application of TENS as a spasticity management modality, this systematic review aims to summarize the research on the use of HF-TENS in the management of PTrS in patients with UMNS.

## Review

Methodology

The prespecified protocol of this systematic review is registered in the Open Search Framework and is available at the following link (doi: 10.17605/OSF.IO/ABP3N). A systematic review of clinical trials, cross-sectional studies, and randomized controlled trials regarding the effectiveness of HF-TENS in patients with pyramidal tract-related lower limb spasticity was performed according to the Preferred Reporting Items for Systematic Review and Meta-analyses (PRISMA) guidelines [5]. Three prominent medical databases, MEDLINE, PubMed, and Scopus, were used in the systematic literature search. Two independent reviewers (AK, VG) searched the databases using search strings that included the following terms: “Transcutaneous Electrical Nerve Stimulation,” “High-Frequency Transcutaneous Electrical Nerve Stimulation,” “Spasticity,” and “pyramidal tract related spasticity.” No date or language filtering was applied. The inclusion criteria included the human adult population (≥18 years) with PTrS. Beyond database records, reference lists were also checked for potentially eligible studies. Randomized control trials, clinical studies, and cross-sectional studies with no date or language filtering were included. The primary and secondary research questions were constructed based on the population, intervention, comparison, and outcome (PICO) strategy: P (adult patients with PTrS), I (HF-TENS), C (difference in time, comparison of HF-TENS protocols versus conventional physical therapy), and O (spasticity levels). TENS parameters included the frequency of stimulation (especially high frequency), as well as pulse duration, application field, and intensity. Spasticity outcomes were measured through evaluation scales (e.g., Modified Ashworth Scale (MAS), Composite Spasticity Scale (CSS), etc.), as well as through electrophysiological parameters (e.g., alteration in Hoffmann's reflex (H-reflex), M-wave, H/M ratio in electromyography, etc.). The prespecified inclusion criteria involved human subjects, published in peer-reviewed journals, and investigating the impact of HF-TENS in managing PTrS. Exclusion criteria included nonoriginal studies (e.g., reviews, meta-analyses, case reports) as well as studies involving animal subjects lacking sufficient data on relevant outcomes.

Results

Literature Search

**Figure 1:**
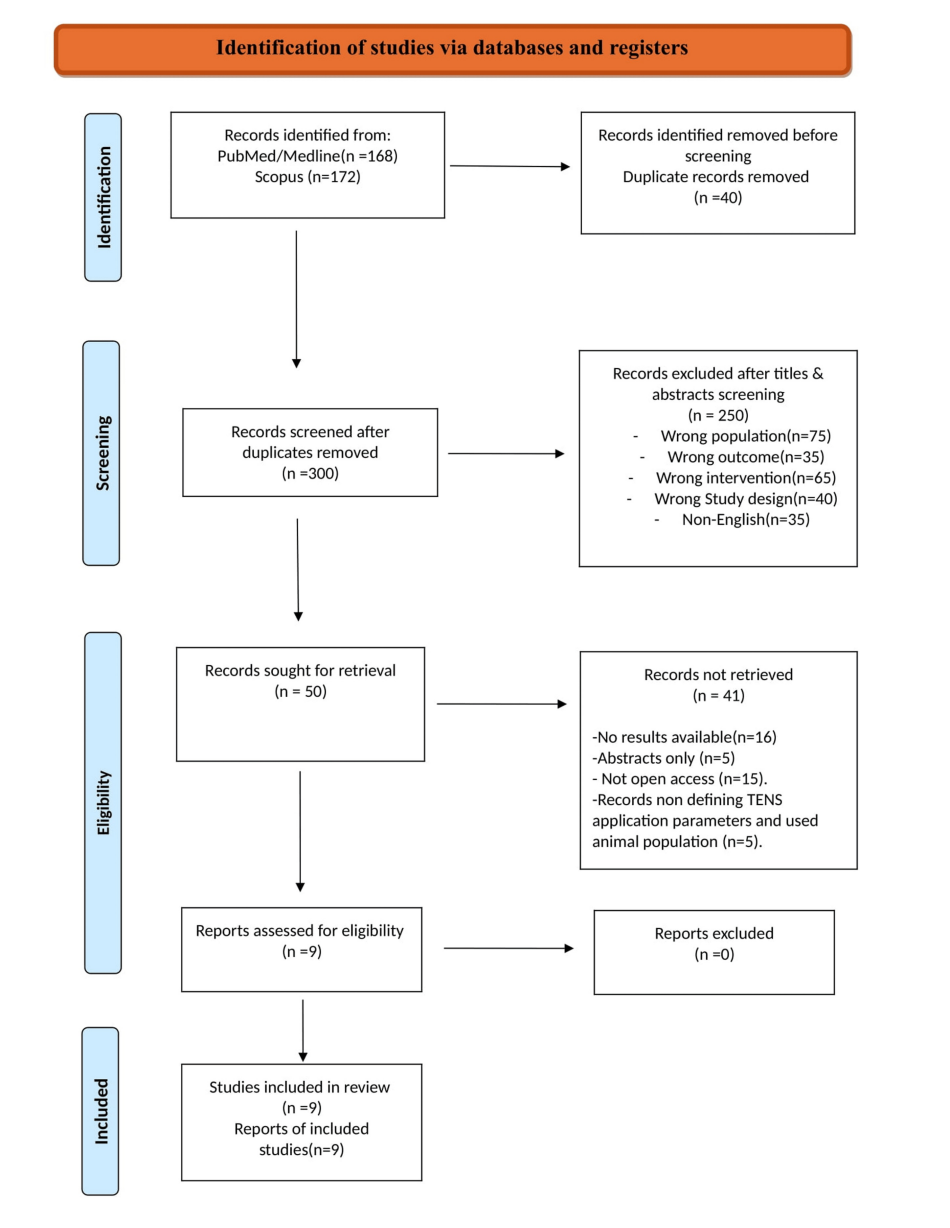
PRISMA flowchart PRISMA: Preferred Reporting Items for Systematic Reviews and Meta-analyses Adapted/reproduced from Page et al. [5], with permission

A total of 340 records were retrieved from the systematic literature search on MEDLINE, PubMed, and Scopus databases. After duplicate exclusion and title and abstract screening, 50 full-text studies were assessed for eligibility. After applying the inclusion/exclusion criteria, 36 studies were excluded from our study, which included articles nonwritten in the English language, systematic reviews, and meta-analyses, as well as parts of books and editorials. Furthermore, five records that do not define TENS application parameters and were performed in an animal population were excluded from our study. As a result, nine records met the inclusion-exclusion criteria and were analyzed [6-14]. The approaches investigated the effectiveness of HF-TENS on pyramidal tract-related lower limb spasticity (Figure 1). Spasticity was measured through the MAS, CSS, and spinal inhibition reflexes through H-reflex (Table 1).

**Table 1:** Descriptive table of the included studies CG; control group; CS: clinical study; CSS: composite spasticity scale; COS: cross-over study; D1: presynaptic inhibition of alpha motor neuron; EG: experimental group; FMA: Fugl-Meyer Assessment; f: frequency; GSS: Global Spasticity Score; HF-TENS: high-frequency transcutanneous electrical nerve stimulation; LF-TENS: low-frequency transcutaneous electrical nerve stimulation; MAS: Modified Ashworth Scale; MS: multiple sclerosis; PSS: Penn Spasm Score; MMT: manual muscle test; PD: pulse duration; RCT: randomized control trial; RI: reciprocal inhibition; SCI: spinal cord injury; SOL: soleus muscle; TA: tibialis anterior muscle; TUGT: timed up and go test

Author	Region	Type of study	N	Age	UMNS	Type of Intervention	Intervention’s duration	Outcome measures	Results
Sonde et al. (2000) ^[6]^	Finland	CS	16	N/a	stroke	HF-TENS: f = 100 Hz; intensity = 0-60 mA; application area: acupuncture point ST 36	30 min, 7 days/week, 3 months total	MAS, FMA, gait time	HF-TENS:↓MAS (p = .01), ↑ gait time (p = .02)
Miller et al.(2007) ^[7]^	Glasgow	COS	32	47 ± 9.8	MS	Group 1, 60 min TENS; Group 2, 8 hrs TENS; PD = 0.125 ms; f = 100Hz	Two weeks	GSS, PSS, VAS	8 hr TENS: ↓ GSS (p = .038), VAS (p = .008)
Cho et al. (2010) ^[8]^	Korea	RCT	26	Placebo TENS (n = 12) 52.17 ± 9.2; HF-TENS (n = 14) 53.07 ± 11.64	Stroke	Placebo TENS HF-TENS f = 100 Hz; PD = 250 μsec 2 times sensory threshold; intensity = 0.01 mA; application area: gastrocnemius muscle	60 min	MMT, MAS, force plate	TENS group: ↓MMT, MAS (p < .05 static balance with eyes open)
Chung et al. (2010) ^[9]^	China	RCT	18	Placebo TENS (n = 10) 50 ± 20; active-TENS (n = 8) 50 ± 20	SCI	HF-TENS f = 100 Hz; PD = 250 μsec; intensity = 15 mA; application area: common peroneal nerve	60 min	CSS	Active TENS: ↓CSS (p = .017), resistance to full ROM (p = .024); ankle clonus (p = .023)
In et al. (2011) ^[10]^	Korea	RCT	26	Placebo-TENS (n = 12) 52.17 ± 9.2; HF-TENS (n = 14) 53.07 ± 11.64	Stroke	Placebo TENS HF-TENS f = 100 Hz; PD = 250 μsec 2 times sensory threshold; intensity = 0.01 mA application area: gastrocnemius muscle	30 min, 5 days/week, 4 weeks total	MAS, handheld manual muscle tester, force plate	HF-TENS: ↓MAS (p < .05 balance strength)
Park et al. (2014) ^[11]^	Korea	RCT	34	Group 1: Therapeutic exercise + placebo-TENS (n = 17) 71.17 ± 3.82; Group 2: Therapeutic exercise + HF-TENS (n = 17) 71.20 ± 3.46	Stroke	Placebo TENS HF-TENS: f = 100 Hz; PD = 200 μsec sensory threshold; intensity = 0.01 mA; application area: gastrocnemius, quadriceps muscle	30 min, 5 days/week, 6 weeks total	MAS, balance system, TUGT, gait analyzer	HF-TENS group: ↓MAS (p < .05 static balance strength, TUGT step length)
Karakoyun et al. (2015)^[12]^	Turkey	RCT	51	Group 1 (n = 24): control 49.88 ± 6.85; Group 2 (n = 27): stroke TENS 60.93 ± 12.8	Stroke + healthy	TENS Group f = 50 Hz; PD = 100 μsec; sensory threshold; intensity = 0-50 mA; application area: tibialis nerve	30 min	MAS, M amplitude, H reflex, Hmax/Mmax, H slope, Hslope/Mslope	TENS group:↓MAS, Hmax amplitude, H/M ratio, Mmax amplitude (p < .05 hmax mmax latencies)
Koyama et al. (2016) ^[13]^	Japan	RCT	20	55.9 ± 9.0	Stroke	HF-TENS: f = 50-100-200 Hz; PD = 250 μsec; ON/OFF time = 40/10sec; intensity = motor threshold: application area: TA, SOL muscles	30 min	Spinal inhibitory reflexes: H-reflex, RI, D1 inhibition	200 Hz TENS ↑D1 inhibition => synaptic transmission from the antagonist to spastic muscles (p = .003)
Lee et al. (2019) ^[14]^	Korea	RCT	20	CG (n = 10): 67.70 ± 4.30; EG (n = 10): 69.60 ± 3.98	stroke	CG: LF-TENS waveform = biphasic symmetrical; f = 3 Hz; PD: 400 μsec; intensity: strong, painful sensations; application area: that gastrocnemius muscle; EG: HF-TENS waveform = biphasic symmetrical; f = 70-130 Hz; PD: 50 μsec; intensity: comfortable tingling or buzzing feeling; application area: gastrocnemius muscle	20 min/day, 5 times/week, total 4 weeks	joint position sense, MAS, TUG	EG group: ↑joint position sense, TUG (p < .05)

Quality Control of the Included Studies

**Figure 2:**
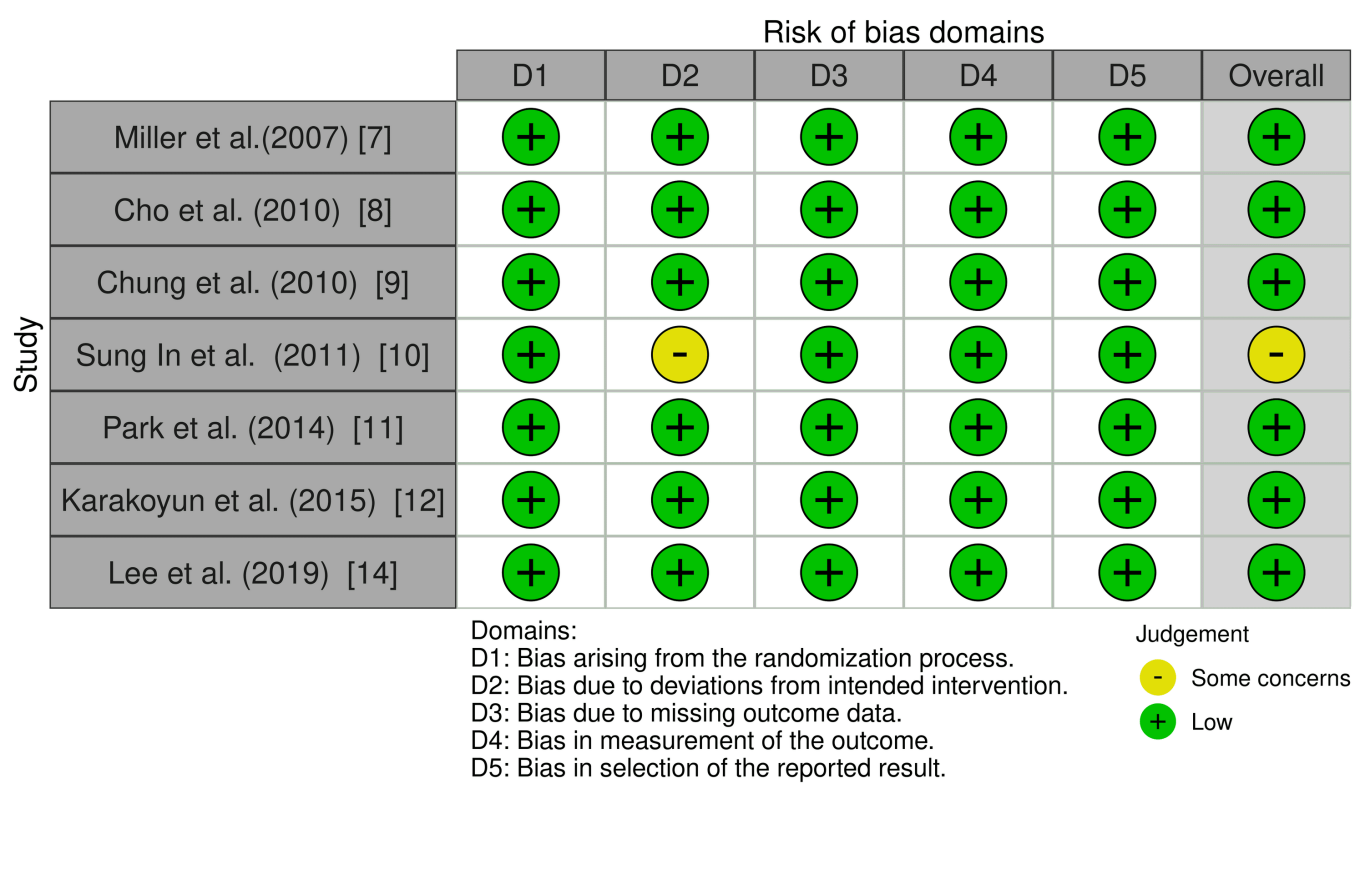
ROBINS-II traffic light plot ROBINS-II: risk-of-bias in randomized studies

**Figure 3:**
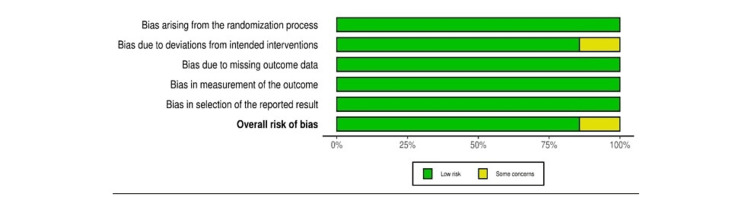
ROBINS-II summary plot ROBINS-II: risk-of-bias in randomized studies

**Figure 4:**
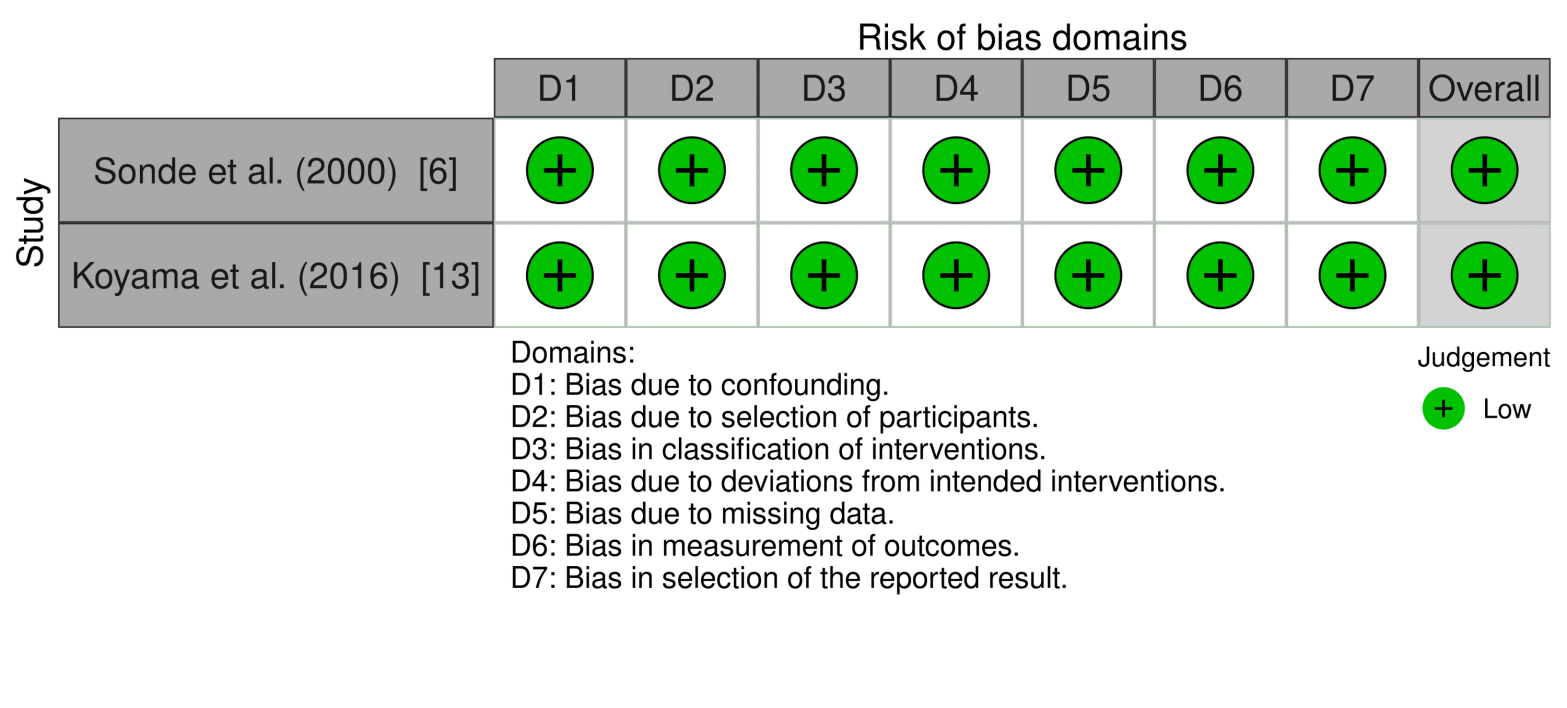
ROBINS-I traffic light plot ROBINS-I: risk-of-bias in nonrandomized studies

**Figure 5:**
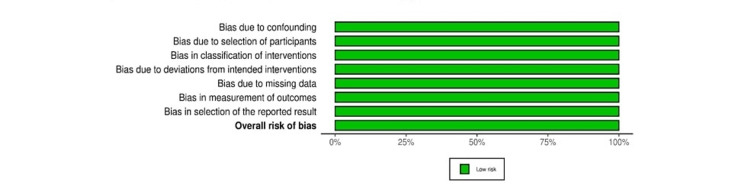
ROBINS-I summary plot ROBINS-I: risk-of-bias in nonrandomized studies

Eligible studies underwent quality assessment using the risk-of-bias in nonrandomized studies (ROBINS-I) and risk-of-bias in randomized studies (ROBINS-II) [15,16]. The overall quality of the included studies was low for eight records and moderate for one record (Figures 2-5).

Clinical Outcomes

Based on the information provided by the included studies, PTrS in humans can be noted into three types of upper motor neuron lesions, reflecting the following neurological conditions: stroke [6,8,10-14], multiple sclerosis (MS) [7], and SCI [9]. Most of the stroke studies compared HF-TENS versus placebo-TENS to examine its effectiveness on lower limb spasticity [6,9,10,13,14]. Application parameters included (1) biphasic symmetrical waveform, (2) 50-200 Hz frequency, (3) intensity set to motor or sensory threshold, (4) application field on gastrocnemius, soleus, tibialis anterior muscles, and (5) duration of 30 to 60 min, for 5-7 days per week for four weeks total [6,8,10-14]. Spasticity’s assessment was performed through the MAS [6,8,10-11,14] or CSS [10], as well as with spinal inhibitory reflexes [13,14]. The results in the stroke population showed that HF-TENS decreased CSS and MAS scores (p < .05). Furthermore, neurophysiological parameters, such as Hmax, Mmax amplitudes, and H/M ratio, were decreased post-TENS treatment (p < .05), whereas Hmax, Mmax latencies, and D1 inhibition were enhanced (p < .05 and p = .03, respectively) [7,9]. In the same spirit as the findings of the aforementioned studies, Lee and colleagues found that the same parameters for HF-TENS, when compared with LF-TENS, reduced lower-limb spasticity levels (p < .05) and increased the lower limb’s range of motion (ROM) and walking speed (p < .05) [6-14].

Despite the results of spasticity in the stroke population [6,8,10-14], Miller and colleagues conducted a cross-sectional study to investigate the effects of spasticity in the MS population. The researchers divided 32 MS patients into two groups receiving HF-TENS with two different application times. In particular, the first study group received HF-TENS for 60 minutes daily, and the second for eight hours daily over a two-week period. The rest of the application parameters were set to 100 Hz frequency and 0.125 ms pulse width. The outcome measures included the Global Spasticity Score (GSS) and visual analogue scale (VAS). Statistical analysis revealed that the eight-hour-HF-TENS group experiences a reduction in spasticity through the decreased muscle spasms (p = .038), as well as in pain levels (p = .008). Similarly, Chung et al. explored the effects of HF-TENS in 18 patients with T12 SCI and bilateral lower limb spasticity. HF-TENS was applied to the common peroneal nerve for 60 minutes, and spasticity was evaluated using the CSS. Their results indicated that electrical stimulation decreased spasticity levels (p = .017), which led to reduced resistance to full ROM (p = .023) and reduced ankle clonus (p = .023) [7,9].

Discussion

To our knowledge, this systematic review is the first to examine the effectiveness of HF-TENS in managing PTrS. More specifically, a total of nine studies encompassing 243 patients with PTrS were included in this systematic review. The phenotype of the upper motor neuron lesions in this study included three types of neurological conditions (stroke, MS, and SCIs). Our findings reported that HF-TENS reduced spasticity levels, as measured by clinical scales and neurophysiological parameters.

Spasticity is the most common clinical sign that is correlated with pyramidal tract lesions. Spasticity’s evaluation includes clinical scales and neurophysiological methods such as electromyography. Especially, H-reflex that is reflected as H-wave, as well as M-wave, and Hmam/Mmax ratio seem to be elevated in spasticity patients. There is a wide spectrum of modalities that could be used for spasticity management, such as pharmacological means (intramuscular injections of botulinum toxin or intraneural phenol injections, intrathecal baclofen pumps, oral administration of baclofen) as well as surgical procedures aimed at modifying the structures of muscles, neurons, or tendons. Additionally, physical therapy, which encompasses techniques such as hydrotherapy, electrical stimulation, and stretching or strengthening exercises, plays a critical role in managing spasticity.

This review highlighted the therapeutic potential of alternative rehabilitation methods, such as electrical stimulation, as valuable complementary interventions. Electrical stimulation can be used either solo or in combination with different physical therapy modalities in order to produce optimal results, taking into consideration the specific needs and limitations of each individual patient. HF-TENS is a noninterventional electrophysical treatment modality that applies electrical current through adhesive electrodes and stimulates large sensory mechanisms. It is usually used to reduce pain in different pathological conditions, by activating pain gait mechanisms through the pain gait mechanism and the endogenous opioid system, respectively. However, TENS’ action mechanism on reducing spasticity has not yet been described. As a noninvasive technique, TENS, particularly at higher frequencies and sensory intensity, was proven effective in alleviating pain, reducing hypertonia, and enhancing muscle endurance without inducing muscle contractions. Taking into consideration the debilitating effects of spasticity on patients' daily functioning and overall quality of life, this review proposes that HF-TENS poses a promising, patient-friendly option in spasticity management [17,18]. Overall, the results indicated that HF-TENS has demonstrated positive effects in reducing lower limb spasticity, as measured by clinical evaluation scales such as the MAS and the CSS, as well as through neurophysiological assessments like spinal inhibitory reflexes. In most neurological conditions, the frequency is set between 50 and 200 Hz, in order to stimulate larger mechanosensitive nerve fibers in the skin [7]. The most commonly used waveform is biphasic symmetrical, while the range of pulse duration of 200-250 μsec may increase the total electrical charge absorbed by the targeted structure, depending on the application time. Moreover, the intensity is set to the sensory threshold, in order to produce a noticeable sensation (i.e., tingling, muscle twitching) without causing pain [18-20]. Finally, the duration of treatment is set from 30 to 60 minutes, for five to seven days per week, for a period of two to four weeks total [6-14].

Based on the spasticity’s evaluation through evaluation scales, several studies have used the CSS to evaluate the effects of high-frequency neuromuscular electrical stimulation (NMES) on the plantar flexor muscles of post-stroke survivors with plantar flexor spasticity. These studies reported significant reductions in CSS scores, with these improvements persisting at the two-week follow-up. Similarly, our previous systematic review found that high-frequency NMES applied to spastic upper and lower limbs in post-stroke survivors significantly reduced spasticity, as measured by MAS, which in turn improved muscle strength and overall patients’ functionality. Furthermore, different forms of electrical stimulation (particularly LF-TENS and interference currents) seem beneficial in improving pain, functional capacity, and quality of life in patients with MS [21-24].

While the exact pathophysiological mechanisms underlying spasticity remain unclear, a possible etiology of spasticity refers to an increase in spinal inhibitory reflexes. After the initial lesion (cerebral or spinal), reflex hyperexcitability, increased muscle stretch reflex due to the loss of inhibitory spinal control, and disynaptic reciprocal inhibition (RI) are most likely to be involved in spasticity in humans [17,21,22]. The knowledge of these spasticity mechanisms has been obtained from electrophysiological studies with alterations in some neurophysiological parameters. Firstly, H-reflex is one of the most commonly used electrophysiological methods in evaluating spasticity and reflects the level of excitability of the alpha motor neuron pool directly associated with the spinal column. Secondly, the M response is the discharge of the alpha motor neurons in the spinal cord of the muscle, with direct stimulation of the muscle efferent nerve. Thirdly, the latency of the H-reflex does not change, but the amplitude and the H/M ratio increase in spasticity. However, some studies demonstrate that the number of inhibitory reflexes is reduced in UMN lesions. According to our findings, HF-TENS, particularly at 200 Hz, reduces spasticity levels by enhancing D1 inhibition and increasing Hmax and Mmax latencies, as well as decreasing Hmax and Mmax amplitudes. However, no statistically significant changes were observed in the RI or H-reflex parameter [22,23,25-30].

Therefore, 200 Hz-HF-TENS appears to improve plasticity of synaptic transmission from the antagonist to the spastic muscles in the UMNS. Takeda and colleagues have yielded similar findings in their study on the effects of TENS on presynaptic inhibition (D1 inhibition) and disynaptic RI in healthy individuals. The researchers demonstrated that, regardless of stimulus frequency, TENS had no effect on RI at any frequency but induced significant changes in D1 inhibition. On the contrary, for the H-reflex, Ridha and colleagues found that 10 Hz TENS application to 31 SCI patients led to positive alterations in the Hmax/Mmax ratio, thereby indicating a reduction in spasticity. In addition to the neuroplastic effects of HF-TENS, a study by Meesen and colleagues found a significant increase in cortical motor representation on the corticospinal projection to the finger and forearm, which can be highlighted through slight increases in motor evoked potentials (MEPs) amplitude. These alterations on MEPs can be reflected with a reduction in spasticity levels [31], highlighting the importance of electrical stimulation as a complementary therapy in neurorehabilitation [32-34].

Finally, in addition to human studies, several animal studies have assessed the efficacy of HF-TENS in animals with bilateral lower limb spasticity after SCI. The experimental rodent models that were used generated contusion SCIs closely paralleling human SCI clinical cases. As a result, these findings could alter the therapeutic potential of HF-TENS in reducing PTrS. In particular, TENS lowers persistent sensitization and nociceptor cell activity in the central nervous system following SCI and when applied to somatic receptive field transection of the chord. Additionally, in rodent models with T10 and T12 SCI and bilateral lower limb spasticity, long-term A-delta activity, generated by TENS, suppresses the activity of central nociceptor cells for up to two hours. Cho and colleagues, following HF-TENS 100 Hz (200 μsec) application to the anterior tubercle of the tibia, showed a significant reduction in spasticity seven days post-treatment compared to the placebo-TENS (control group), as measured by MAS (p < .05). In addition to the above study, Hahm et al. further compared the effects of LF-TENS and HF-TENS on spasticity, using three different intensities (motor threshold (MT), 90% MT, and 50% MT). The researchers assessed spasticity via MAS and microglia activation via immunohistochemistry, revealing that HF-TENS at 90% MT resulted in significant reductions in MAS scores and activated microglia (p < .001) [25,35].

Hence, the authors may conclude that HF-TENS may be a valuable adjustive tool that could be used in neurorehabilitation. Despite its analgesic role, HF-TENS could be beneficial in managing PTrS. However, the high heterogeneity of application parameters highlights the need for stratifying a therapeutic protocol for spasticity management. According to our findings, a possible HF-TENS for spasticity management may include the following parameters: frequency, 50-200 Hz; waveform: biphasic symmetrical, respectively; pulse duration range from 200 to 250 μsec; and the treatment duration is set on 30 to 60 minutes for five to seven days per week, for four weeks total.

This study is not without limitations. To begin with, there was a heterogeneity in upper motor neuron lesions included in the study, which enlarged the research strategy and the inclusion criteria for this systematic review. However, spasticity is defined as a clinical symptom that occurs after upper motor neuron lesions, which is differentiated from spasticity that is associated with cerebral palsy. As a result, we focused on adult patients with PTrS. Additionally, there is a high degree of heterogeneity among the application parameters, due to the variation in intervention protocols and outcome measures, which may have contributed to the observed variability of the results. Despite the abovementioned methodological limitations, the results of the nine studies were significant, reporting the effect sizes in all cases, and highlighted a beneficial effect of HF-TENS on patients with PTrS.

## Conclusions

HF-TENS is a common therapeutic modality used by physical therapists in a wide range of musculoskeletal and neurological rehabilitation protocols, including spasticity management. This systematic review aimed to summarize the current intervention protocols of HF-TENS for patients with PTrS. The findings demonstrated the beneficial effects of HF-TENS on reducing spasticity, as well as improving both static and dynamic balance. However, the heterogeneity of HF-TENS in different application parameters prevented the establishment of clear guidelines, emphasizing the need for protocol standardization. Based on the literature review and clinical experience, the frequency and waveform used for the management of PTrS are commonly set in the specified range (50-200 Hz and biphasic symmetrical, respectively). Additionally, pulse duration ranges from 200 to 250 μsec, and the treatment duration is set at 30 to 60 minutes for five to seven days per week, for four weeks total. Future studies should focus on stratifying an HF-TENS protocol in order to avoid any negative impact on patients with PTrS. Lastly, it would be beneficial to maintain the homogeneity of all electrical application parameters and the heterogeneity of the sample in the domains of gender, age, and socioeconomic status.
